# SE-MAConvLSTM: A deep learning framework for short-term traffic flow prediction combining Squeeze-and-Excitation Network and Multi-Attention Convolutional LSTM Network

**DOI:** 10.1371/journal.pone.0312601

**Published:** 2024-12-05

**Authors:** Rong Zhu, Jie Tang, Xuansen He, Xianlai Zhou, Xiaohui Huang, Fengyun Wu, Songli Chen

**Affiliations:** 1 School of Modern Information Industry, Guangzhou College of Commerce, Guangzhou, China; 2 Guangdong Embedded Information Technology Industry College, Guangzhou Xinhua University, Guangzhou, China; 3 School of Information Engineering, East China Jiaotong University, Nanchang, China; New York University Abu Dhabi, UNITED ARAB EMIRATES

## Abstract

Traffic flow prediction is an important part of transportation management and planning. For example, accurate demand prediction of taxis and online car-hailing can reduce the waste of resources caused by empty cars. The prediction of public bicycle flow can be more reasonable to plan the release and deployment of public bicycles. There are three difficulties in traffic flow prediction to achieve higher accuracy. Firstly, more accurately to capture the spatio-temporal correlation existing in historical flow data. Secondly, the weight of each channel in the traffic flow data at the same time interval affects the prediction results. Thirdly, the proportion of closeness, period and trend of traffic flow data affects the prediction results. In this paper, we design a deep learning algorithm for short-term traffic flow prediction, called SE-MAConvLSTM. First, we designed Spatio-Temporal Feature Extraction Module (STFEM), which is composed of Convolutional Neural Network (CNN), Squeeze-and-Excitation Network (SENet), Residual Network (ResNet) and Convolutional LSTM Network (ConvLSTM) to solve the above two problems mentioned. In addition, we design multi-attention modules (MAM) to model the closeness, period and trend of traffic flow data to solve the third problem mentioned above. Finally, the aggregation module was used to integrate the output of the last time interval in STFEM and the output of the multi-attention module. Experiments are carried out on two real data sets, and the results show that the proposed model reduces RMSE by 4.5% and 3.7% respectively compared with the best baseline model.

## 1 Introduction

In recent years, the concepts of intelligent transportation systems and smart cities have been repeatedly mentioned, and traffic flow prediction is one of the important research topics. Accurate prediction of future traffic flow can help traffic management departments to provide the best driving routes for travelers, balance traffic flow, and optimize traffic travel structure, so as to solve traffic congestion problems, improve road safety, and make up for the development of urban travel service system. At present, the advancement of urbanization makes the transportation network increasingly complex, and the demand of travelers in public transportation connection, daily commuting, business and leisure is also showing the characteristics of high quality, diversity, personalization and low carbon, which puts forward higher accuracy requirements for real-time dynamic prediction of traffic flow. Traffic flow prediction is to predict the future traffic flow of each area of the city according to the past traffic flow distribution, in essence, it is a spatio-temporal sequence prediction problem. Ideally, from the perspective of the time dimension, the traffic flow at the current time is necessarily affected by the traffic flow at the previous time period, for example, the growth of the traffic flow at the previous time period can lead to the growth of the traffic flow at the next time period. From the perspective of spatial dimension, the traffic flow in a certain area of the city is inevitably affected by the traffic flow in the surrounding area. For example, a large influx of vehicles in the nearby area can lead to the growth of the traffic flow in the central area, and the growth of the traffic flow in the central area will cause the increase of the vehicle outflow flow in the next period. It can be seen that the challenges of accurately predicting future traffic flow are as follows:

1. Extract temporal dependencies and spatial dependencies between adjacent regions from historical traffic flow data.
2. The influence of the ratio of each channel in the data on the prediction results in the same time period.
3. Accurately capture the unique properties of traffic flow, including closeness, period and trend.

In order to capture the above features more accurately, we propose a deep learning framework called SE-MAConvLSTM, which combines Squeeze-and-Excitation Network and Multi-Attention Convolutional LSTM Network. The main contributions of this paper are as follows:

1. This paper proposes a deep learning framework for traffic flow prediction, which can effectively obtain a series of potential features of historical traffic data.
2. In order to address the first and second challenges, this paper designs a Spatio-Temporal Feature Extraction Module (STFEM), which can effectively obtain spatio-temporal dependencies. The SENet module in STFEM uses the attention mechanism, which can help the model automatically assign weights to each channel in the same time interval data during the training process.
3. To address the third challenge, we design a multi-attention module (MAM) to capture the proximity, periodicity and trend in traffic flow data, and it assign large weights to historical data similar to the predicted data in the training process.
4. Experimental results on two datasets show that SE-MAConvLSTM algorithm exhibits state-of-the-art performance, improving the performance by 4.5% and 3.7% respectively compared with the best baseline algorithm on two datasets.

The rest of this paper is organized as follows. Section 2 provides a detailed description of the research background and related work. Section 3 gives a definition of traffic flow prediction. Section 3 specifically describes the SE-MAConvLSTM framework structure. Section 4 provides a detailed analysis of the datasets, hyperparameters and metrics, baseline algorithms, model training, and experimental procedures in the proposed model. Section 5 summarizes the SE-MAConvLSTM framework and gives an outlook on future work.

## 2 Related work

As an important part of smart city, urban traffic flow prediction has always been a research hotspot. At present, many scholars have fully studied the related work. It consists of four stages:

### 2.1 Traditional models based on time series

Rezzouqi et al. [1] applied HA method to predict urban traffic flow. Autoregressive Integrated ARIMA [2] are used to capture periodicity in traffic flow. Abanto et al. [3] used ARIMA to model the traffic behavior in peak hours and detect abnormal behaviors such as traffic accidents. Kumar et al. [4] improved ARIMA and proposed a model called SARIMA for traffic prediction. Because the model based on time series does not consider the relationship with space, the prediction accuracy is low when the road section is complex and the traffic flow data changes dramatically.

### 2.2 Models based on machine learning

Zhan et al. [5] proposed a hybrid framework based on Bayesian network and graph model to improve the accuracy of traffic flow prediction. Liang et al. [6] proposed a method combining Kalman filter and k-nearest neighbor to predict short-term passenger travel flow. Chen et al. [7] proposed a dynamic cluster-based demand prediction framework to predict the flow of station-based shared bicycles. Kwon et al. [8] proposed a CHMM to predict the traffic flow on highways, and further evaluated the effectiveness of the high-occupancy vehicle system. The capacity of the model based on machine learning is limited, and it cannot mine the periodicity of traffic flow data well. The time overhead is large, and the prediction accuracy of the traffic flow data with large amount of data and many influencing factors is not high.

### 2.3 Spatio-temporal sequence model based on deep learning

Zhang et al. [9] proposed a prediction model DeepST based on deep learning for urban spatio-temporal data to predict urban traffic flow, which considered both spatial dependence (such as geographic hierarchy and distance attributes) and temporal dependence (such as proximity, periodicity, trend) of cities. Zhang et al. [10] also proposed a model named ST-ResNet, which uses residual network to model the proximity, periodicity and trend of crowd flow. By dynamically aggregate the output of three residual neural networks, different weights are assigned to different time attributes and regions. At the same time, it further combines external factors such as weather to predict the final flow of people in each area. Shen et al. [11] proposed StepDeep framework to fully encode the dependence between space and time by using temporal convolution filter, spatial convolution filter and spatio-temporal convolution filter. Yuan et al. [12] proposed a Hetero-ConvLSTM framework based on ConvLSTM model to predict urban traffic accidents. This method solves the problem that traffic accidents are rare in time and heterogeneous in space. Wu et al. [13] proposed a traffic flow prediction model DNN-BTF based on deep neural network. The model makes full use of the periodic characteristics and spatio-temporal characteristics of traffic flow, and introduces an attention mechanism, so that the model can automatically learn the importance of historical traffic flow at different times. Lv et al. [14] first proposed a prediction model based on an autoencoder module to predict urban traffic. This method used a stacked autoencoder model to learn general traffic flow features and trained in a greedy hierarchical manner. Although the model based on deep learning can achieve good traffic flow prediction effect, in addition to periodicity, there will be random errors in traffic flow. To eliminate this random error, a large number of different types of data are needed for supplement, such as multiple traffic flow parameters.

### 2.4 Attention-based models

Woo et al. [15] proposed CBAM (Convolutional Block Attention Module), which can automatically select and strengthen important features in space by introducing an attention mechanism to learn the relationship between channels and spatial position relationship, and improve the performance of the network. Lin et al. [16] proposed a memory-based self-attention model to memorize the global spatio-temporal dependencies in the prediction process and improve the accuracy of traffic flow prediction. Lin et al. [17] proposed a Multi-aspect attention (MAA) model, which applies scale dot product attention to the entire spatio-temporal information. Yu et al. [18] obtain the dependence between data through the multi-head self-attention mechanism, input the dependence into the encoder-decoder multi-attention mechanism module, and use the self-attention mechanism to suppress the loss of long-term time series information and effectively improve the prediction accuracy. Although the Self-Attention Mechanism is good at capturing the internal correlation of data or features, and the multi-head self-attention mechanism can simultaneously identify multiple subspace information at different locations, it lacks the location and time information of spatio-temporal features, and cannot distinguish the influence between different spatio-temporal features. Yan et al. [19] proposed a deep learning framework called ProSTformer, which uses self attention mechanisms in both spatial and temporal dimensions. It can achieve spatial dependency from local to global, as well as temporal dependency from internal to external. Cai et al. [20] proposed a traffic flow prediction method based on an encoder decoder framework. It uses GCN module and Transformer module respectively to capture the spatial and temporal features of traffic data. Chen et al. [21] proposed a Bi STAT framework consisting of spatial adaptive Transformer, temporal adaptive Transformer, and DHM module to address the issue of uneven temporal and spatial distribution in traffic flow data.

In general, in the current research, most researchers use some methods based on attention to solve the temporal and spatial dependencies.

## 3 Definition

### 3.1 Division of urban areas

**Fig 1:**
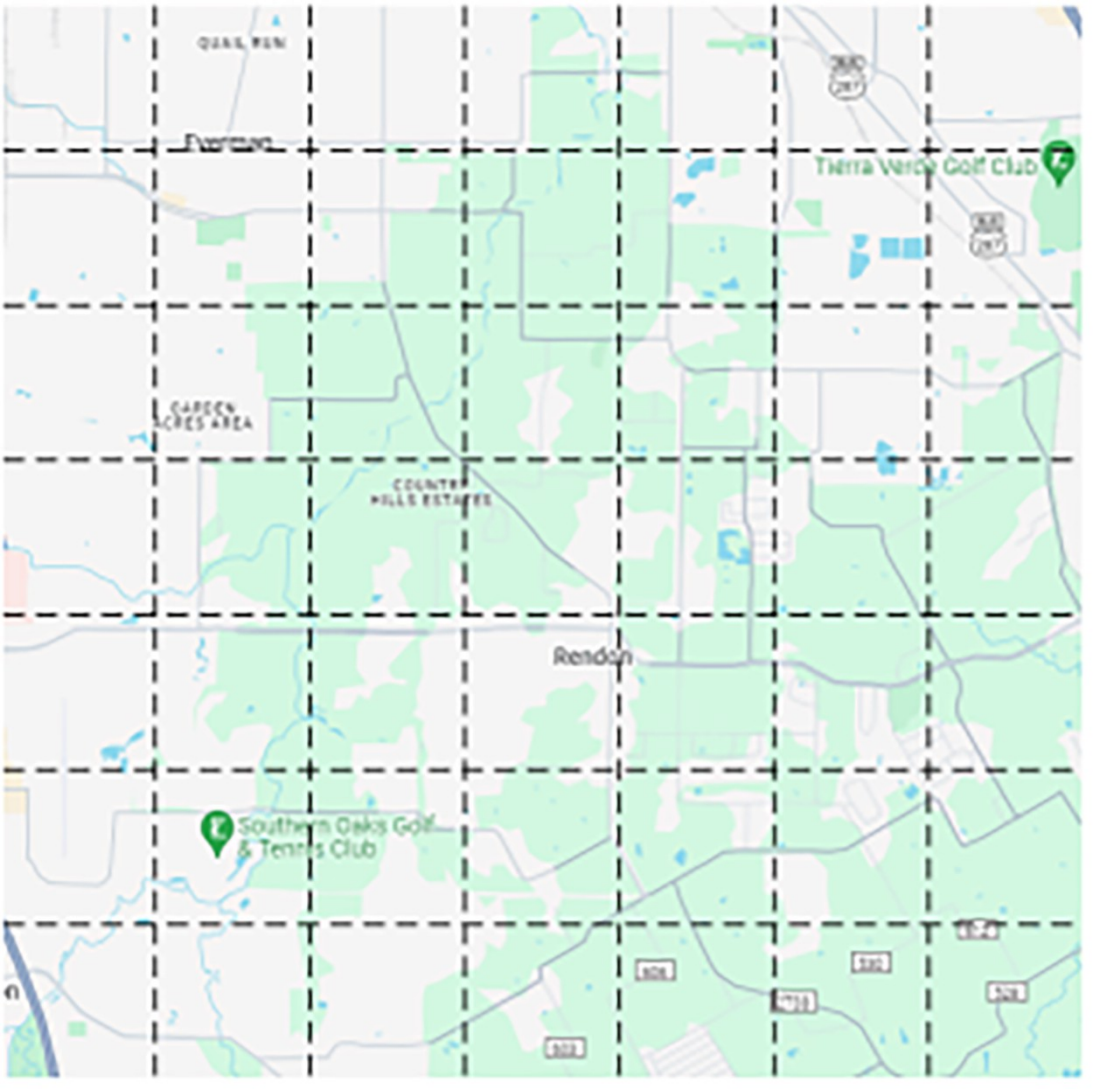
Grid map.

We adopt a gridding approach based on the earth’s latitude and longitude coordinate system, using step size *L* to divide the city map into *m* × *n* non-overlapping geographic grids, as shown in Fig 1. Each grid represents a region, and the *i* row and *j* column of the region are denoted by *R*_*ij*_, and the set of all regions is defined as *R* = {*R*_*ij*_∣*i* ∈ (1, 2, …, *m*), *j* ∈ (1, 2, …, *n*)}, as shown in Fig 1.

### 3.2 Inflow matrix/Outflow matrix

**Fig 2:**
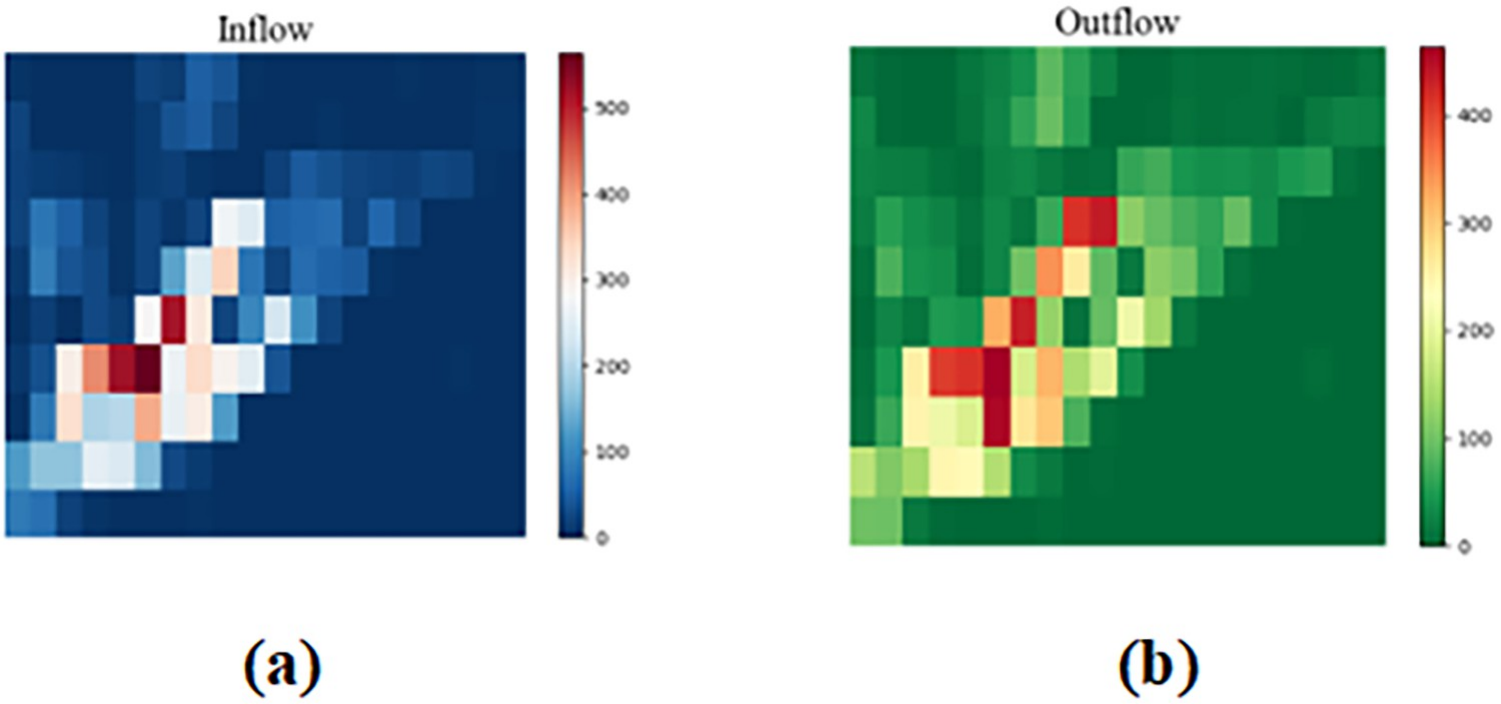
An example of inflow matrix and outflow matrix. (a)Inflow Matrix (b)Outflow Matrix.

Given the time interval *t*^*th*^ of the data, the traffic inflow of region *R*_*ij*_ is the sum of the traffic flows into *R*_*ij*_ from other regions during the time interval. The traffic outflow of region *R*_*ij*_ is the sum of the traffic flows from *R*_*ij*_ to other regions in the time interval. For the time interval *t*, all regions adopt the same way, and we can obtain the traffic inflow matrix and traffic outflow matrix for the time interval *t*. Each region contains inflow and outflow two channels, and the traffic flow in each time period can be represented as a tensor *X* ∈ *R*^2×*m*×*n*^. As shown in the Fig 2, it is the inflow matrix and outflow matrix of New York City for a given time period.

### 3.3 Traffic flow prediction

This paper predicts the traffic flow $\hat{X}$ at a certain time in the future. The input data uses the trend data *X*^*Trend*^, the period data *X*^*P*^, and the closeness data *X*^*C*^. For example, we predict time interval *t* + 1 on day *T*, $X_{t+1}^{day=T}$, the trend data input is $X^{Trend}=\left\{X_{t}^{T-7},X_{t+1}^{T-7},X_{t+2}^{T-7}\right\}$, the period data input is $X^{P}=\{\left\{X_{t}^{T-3},X_{t+1}^{T-3},X_{t+2}^{T-3}\right\},\left\{X_{t}^{T-2},X_{t+1}^{T-2},X_{t+2}^{T-2}\right\},\left\{X_{t}^{T-1},X_{t+1}^{T-1},X_{t+2}^{T-1}\}\right\}$, the closeness data input is $X^{C}=\left\{X_{t-9}^{T},X_{t-8}^{T},...,X_{t}^{T}\right\}$.

## 4 The SE-MAConvLSTM framework

**Fig 3:**
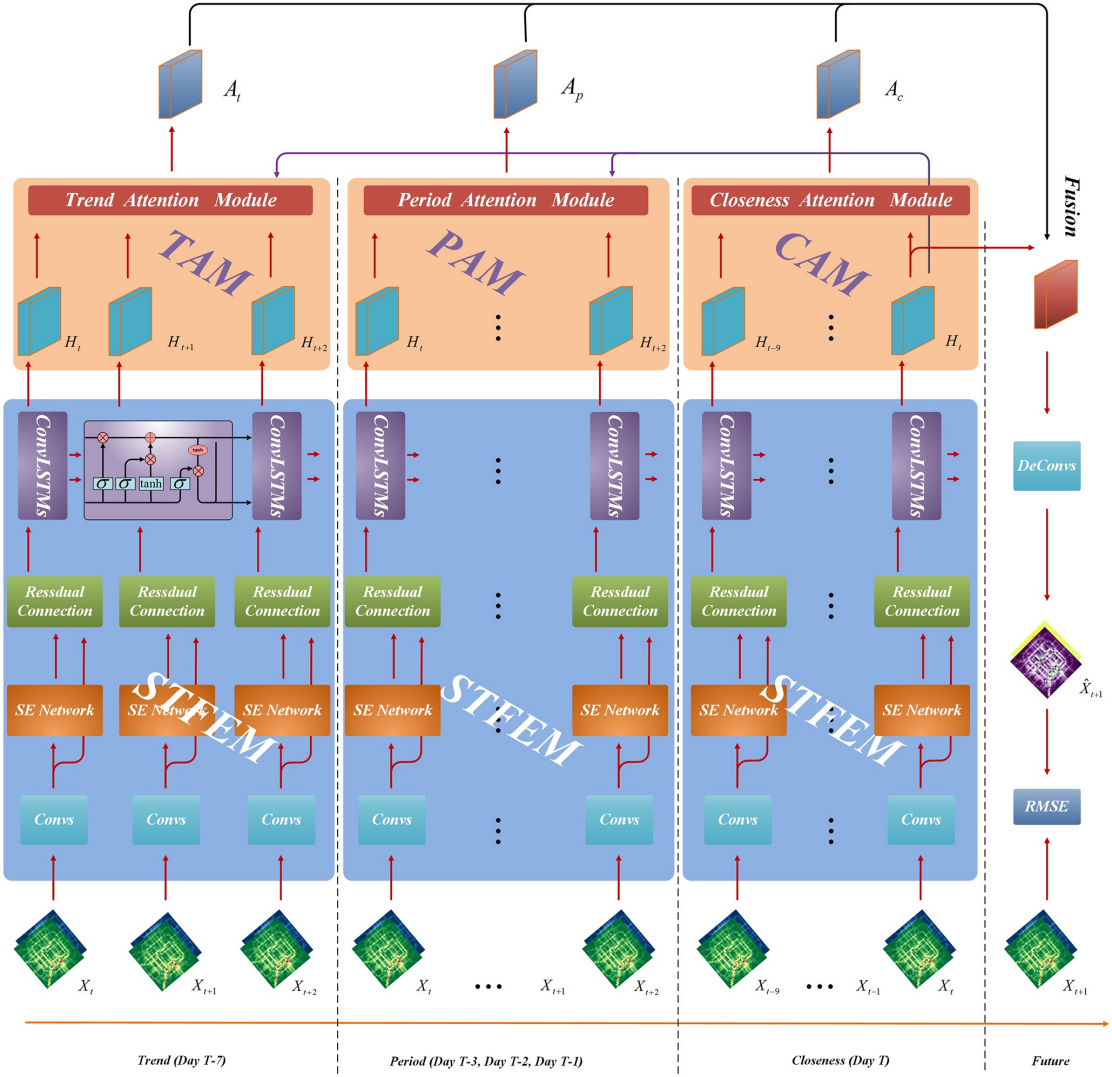
SE-MAConvLSTM framework.

Fig 3 shows the overall architecture of SE-MAConvLSTM framework, which is mainly composed of three parts: Spatio-temporal Feature Extraction Module (STFEM), Multi-Attention Module (MAM) and Fusion Module.

### 4.1 STFEM

**Fig 4:**
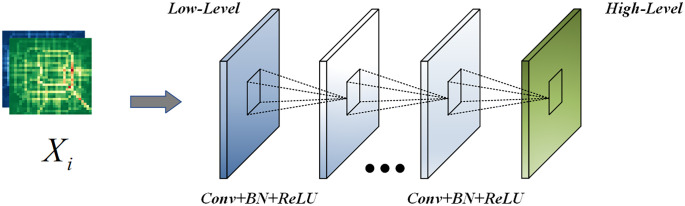
Multi-convs.

First, the input data *X*_*i*_ is passed through a multi-layer convolutional neural network, which is mainly used to capture the spatial features between different regions, and the ReLu activation function is used after each convolution. In this way, we can obtain for each output *I*_*i*_ of the input data, as shown in Fig 4.

$$\begin{array}{c} I_{i}=Multi\_Convs\left(X_{i}\right) \end{array}$$ (1)

**Fig 5:**
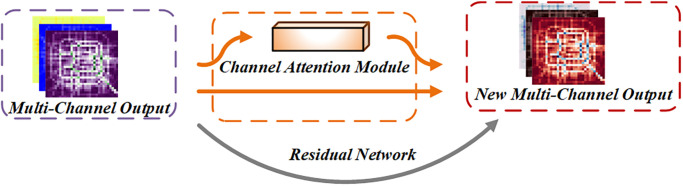
SE Network and Residual Network.

Secondly, the output data *I*_*i*_ is used as the input of the SE Network and the Residual Network. This module is mainly used to automatically assign the weight of the proportion for each data channel, as shown in Fig 5.

Adaptive Average Pooling was performed on data *I*_*i*_ according to spatial dimension, and then one-dimensional vector was obtained by two fully connected networks (FC) learning. Finally, the Channel Attention Vector is multiplied with the original feature correspondence to obtain the attention feature map, and then the residual connection is performed with the original feature.

Finally, the data of the same day were fed into the ConvLSTM network respectively to obtain the spatio-temporal features between the data. ConvLSTM is similar to LSTM. The difference between ConvLSTM and LSTM is that the input of LSTM is a vector and matrix multiplication is used in subsequent operations. However, the input of ConvLSTM network is image-like data, and convolution operation is used in subsequent operations. Both use the same specific formula, but the operation is different. The formula is as follows:

$$\begin{array}{c} i_{t}=\sigma \left(W_{xi}*{Out}_{t}+W_{hi}*H_{t-1}+b_{i}\right) \end{array}$$ (2)

$$\begin{array}{c} f_{t}=\sigma \left(W_{xf}*{Out}_{t}+W_{hf}*H_{t-1}+b_{f}\right) \end{array}$$ (3)

$$\begin{array}{c} o_{t}=\sigma \left(W_{xo}*{Out}_{t}+W_{ho}*H_{t-1}+b_{o}\right) \end{array}$$ (4)

$$\begin{array}{c} C_{t}=f_{t}⊙C_{t-1}+i_{t}⊙\text{tanh}\left(W_{xc}*{Out}_{t}+W_{hc}*H_{t-1}+b_{c}\right) \end{array}$$ (5)

$$\begin{array}{c} H_{t}=o_{t}⊙\text{tanh}\left(C_{t}\right) \end{array}$$ (6)

where *Out*_*t*_ is the input of the ConvLSTM network, *H*_*t*−1_ is the hidden state at the *t*_1_ time interval, *W* and *b* are the learning parameters, * denotes the convolutional operation, ⊙ denotes the Hadamard product.

### 4.2 Multi-attention module

After the data *X*_*i*_ passes through STFEM, the trend data can be obtained as {*H*_*t*_, *H*_*t*+1_, *H*_*t*+2_}, corresponding to day *T* − 7, and the periodic data can be obtained as {*H*_*t*_, *H*_*t*+1_, *H*_*t*+2_}, corresponding to day *T* − 1, *T* − 2, *T* − 3, closeness data can be obtained by {*H*_*t*−9_, …, *H*_*t*−1_, *H*_*t*_}, corresponding to day *T*.

**Fig 6:**
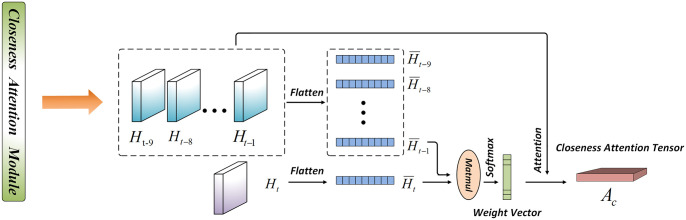
Closeness attention.

As shown in Fig 6, first, the data {*H*_*t*−9_, …, *H*_*t*−1_, *H*_*t*_} stretch into one-dimensional vectors using Flatten and then use {*H*_*t*−9_, …, *H*_*t*−1_} and {*H*_*t*_} respectively perform matrix multiplication to obtain one-dimensional vectors, and then use Softmax operation to normalize them to obtain proximity attention vectors, and finally with the original features {*H*_*t*−9_, …, *H*_*t*−1_, *H*_*t*_} corresponds to multiplication and addition to obtain the closeness attention tensor. The formula is as follows:

$$\begin{array}{c} \alpha_{k}={\bar{H}}_{t}\times {\left({\bar{H}}_{k}\right)}^{T},\forall k\in \left[t-9,t-1\right] \end{array}$$ (7)

$$\begin{array}{c} {\bar{\alpha }}_{k}=\frac{\text{exp}(\alpha_{k})}{\Sigma_{k=1}^{t-1}\;\text{exp}\left(\alpha_{k}\right)},\forall k\in \left[t-9,t-1\right] \end{array}$$ (8)

$$\begin{array}{c} A_{c}=\sum_{k=t-9}^{t-1}{\bar{\alpha }}_{k}H_{k} \end{array}$$ (9)

where ()^*T*^ is the transpose.

Among them, the periodic and trend attention tensors are acquired in a similar way as proximity.

### 4.3 Fusion module

The Fusion module fuses the output of the three attention modules (CAM, PAM and TAM) with the output *H*_*t*_ of the last time interval *t* in the STFEM module, and inputs the fused data into the transposed convolution for dimension transformation. The predicted value of traffic flow ${\hat{X}}_{t+1}$ for the next time interval *t* + 1 is obtained.

## 5 Experiment

In this section, we elaborate on the overall process of the experiment from the following aspects: dataset, hyperparameters and metrics, baseline model and analysis of the experiment process.

### 5.1 Dataset

In the present paper, we use two datasets, TaxiNYC and BikeNYC, in New York city.

TaxiNYC: In this dataset, New York city is divided into 10 × 20 grid maps, and traffic inflow and traffic outflow are calculated at 30-minute intervals in each cell grid. The selected data sampling time is 60 days from January 1, 2015 to March 1, 2015, of which 40 days are used as the training set, 10 days as the validation set and the remaining 10 days as the test set.

BikeNYC: In this dataset, New York city is divided into 21 × 12 grid maps, and traffic inflow and traffic outflow are calculated at 1-hour intervals in each cell grid. The selected data sampling time is 6 months from April 1, 2014 to September 30, 2014, of which 4 months are used as the training set, 1 month is used as the validation set and the remaining 1 month is used as the test set.

### 5.2 Hyperparameters and metrics

The model was built using the PyTorch framework. Due to the large number of matrix operations involved in the training process, the GPU of 8GB NVIDIA GeForce RTX 3070 Ti was used to speed up the model training. The hyperparameters set for the experiment are as follows:

1. The convolutional layer consists of two layers, the number of filters is 8 and 16, the kernel size is all 3 × 3, the stride value is 1, and the padding is 1. After each convolution layer, a linear Rectifier function (ReLU) is added to map the data.
2. ConvLSTM uses two layers, the hidden state dimension is set to 64, and the kernel size is set to 3 × 3.
3. The deconvolution layer consists of two layers, and the filters are set to 8 and 2, respectively. Other parameters are the same as those of the convolutional layer.
4. Reduction is set to 4 in the SE Network module.
5. The epochs of the two datasets are 800 (TaxiNYC) and 500 (BikeNYC). The batch size is 16 and the experiments use Adam as the optimizer with a learning rate of 0.0001.

In this paper, RMSE (Root Mean Square Error) is used to measure the performance of the model.

### 5.3 Baseline methods

In this experiment, we use seven baseline algorithms for comparison, as follows:

1. HA(History Average Model) [22]
  The weighted average of the historical observations is used as the predicted value. For example, assuming that the observed traffic flow in a certain road segment is over a period of four weeks, the prediction for this Monday is the calculation of the average traffic flow over the last four Mondays.
2. ARMA (Auto-Regressive Moving Average Model) [23]
  ARMA model is a stochastic time series model, which can identify the structure of time series and find the optimal prediction value by minimizing the covariance matrix. It uses the lag series and random disturbance term of time series itself to describe the development law of time series.
3. Copyyersterday
  It uses the data of yersterday as the predicted value.
4. CNN (Convolutional Neural Networks)
  Predicting future values by capturing spatial features of historical observations. The global shared convolution kernel is used to extract and mine the local spatial features of the traffic flow sequence to establish the adjacency relationship between upstream and downstream road segments, which cannot fully represent the location relationship between road segments on complex roads.
5. ConvLSTM(Convolutional LSTM Network) [24]
  Uses temporal and spatial dependencies of historical observations to predict future values. The linear manipulation of one-dimensional data of the original LSTM structure is converted into a convolution operation on two-dimensional data, which outputs a three-dimensional tensor, where the first two dimensions are spatial dimensions and the latter dimension is time dimension, so that the spatial characteristics and time series characteristics of traffic data can be captured at the same time.
6. ST-ResNet(Deep Spatio-Temporal Residual Network) [10]
  Integrate convolutional neural networks and residual connections to capture spatial dependencies of historical data. A residual neural network framework is used to model the time, distance, period and trend properties of traffic flow. A convolution-based residual network is used to model the near-far spatial dependencies between any two regions in the city, while ensuring that the prediction accuracy of the model is not affected by the deep structure of the neural network. It has better performance in node traffic prediction.
7. PCRN(Periodic Convolutional Recurrent Network) [25]
  ConvGRU and attention mechanism are used to capture the latent dependencies of historical observations. The periodic Convolutional Recurrent Network uses a convolutional recurrent network to capture the spatio-temporal representation, and fuses the periodic representation, the current input sequence representation, and the temporal metadata.
8. DeepSTN+(Context-Aware Spatio-Temporal Neural Network) [26]
  an improved version of the ST-ResNet algorithm, in which a new ConvPlus module is used to obtain spatial features of historical observation data. The ConvPlus structure is used to model the long-distance spatial dependencies of different regions, and the time factor is combined to express the influence of location attributes.

### 5.4 Analysis of experimental results

#### 5.4.1 Model training process

**Fig 7:**
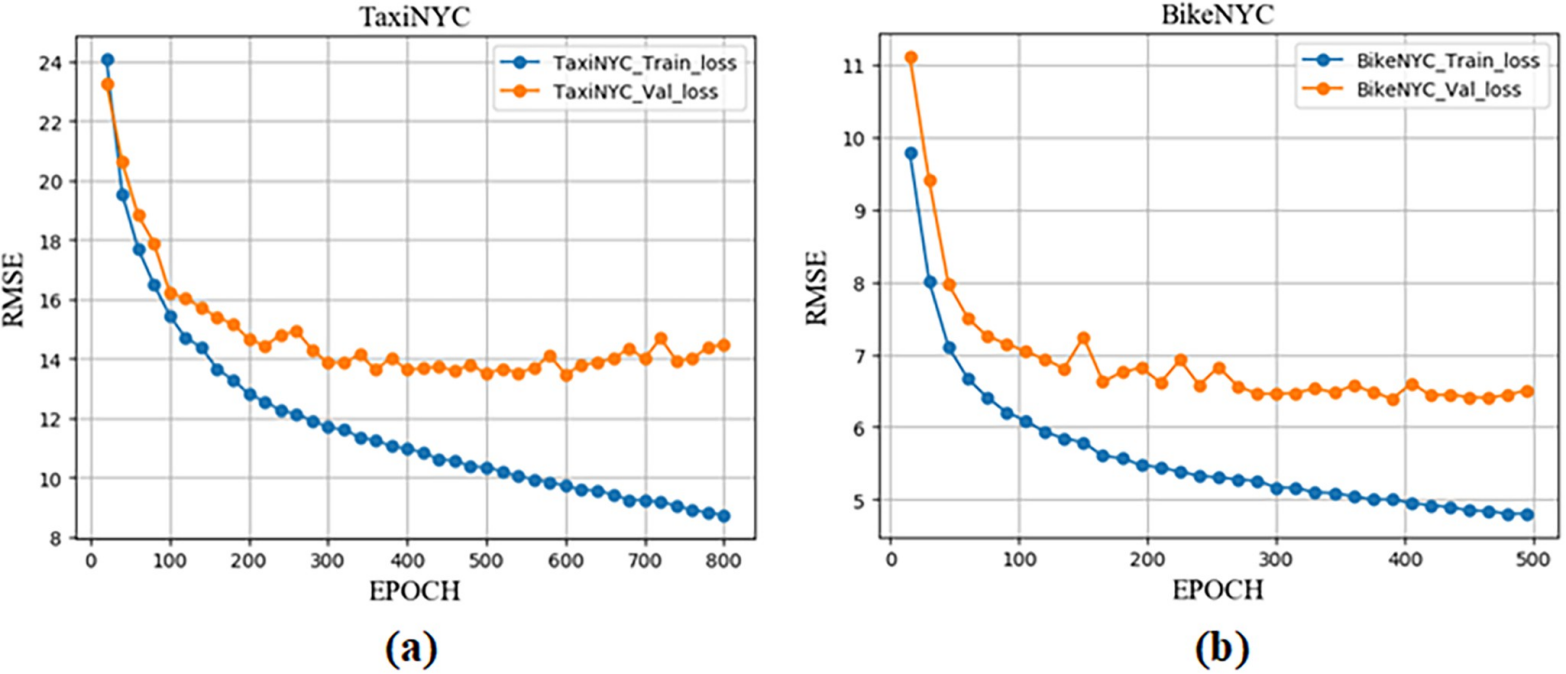
Training and validation loss process of SE-MAConvLSTM on two datasets. (a)TaxiNYC (b)BikeNYC.

The training phase (including training set process and validation set process) of the SE-MAConvLSTM algorithm in dataset TaxiNYC and dataset BikeNYC is shown in Fig 7(a) and 7(b). It can be seen from the figure that the RMSE value of our proposed algorithm on the training set gradually decreases, and convergence is achieved on the validation set. Based on the results of the training and validation sets, the EPOCH values are set to 537 and 310 for the two datasets, respectively.

#### 5.4.2 Comparison of experimental results

Firstly, the comparison between the results of the model trained on the two data sets and other benchmark models is given, as shown in Table 1.

**Table 1:** Comparison of the trained results with other baseline models.

Model	TaxiNYC	BikeNYC
HA	21.833	15.989
ARMA	17.232	16.518
Copyyesterday	18.453	14.640
CNN	16.881	12.651
ConvLSTM	12.533	6.821
ST-ResNet	12.043	6.542
PCRN	13.032	11.303
DeepSTN+	12.010	6.727
SE-MAConvLSTM (ours)	**11.475**	**6.302**

As can be seen from Table 1, our proposed SE-MAConvLSTM algorithm achieves the best performance on both datasets, with RMSE values of 11.475 and 6.302, respectively, which is 4.5% and 3.7% lower on the two datasets, respectively, compared with the best effect among the benchmark algorithms. Traditional algorithms including HA, ARMA, Copyyesterday, as a whole, their effect is relatively poor, indicating that it is difficult to obtain the potential complex rules of traffic flow data. Copyyesterday is slightly better than HA and ARMA, indicating that the closer to the predicted data, the more it shares a certain degree of similarity.

Deep learning methods include CNN,ConvLSTM,ST-ResNet,PCRN. As can be seen from the table, the effect of deep learning algorithm is better than the traditional algorithm on the whole, which indicates that deep learning has great advantages when dealing with more complex nonlinear data. The performance of ConvLSTM is significantly better than that of CNN (25.8%/46.1% for TaxiNYC/BikeNYC) because of more temporal features. Among the benchmark algorithms, ST-ResNet and DeepSTN+ perform best on the two data sets, respectively. Their main components are the use of CNN and residual connection, and the capture of the potential characteristics of traffic data (closeness, period, trend), which also shows the effectiveness of the above modules for learning the potential features of traffic data. PCRN borrows the construction idea of pyramid in image domain, and the performance improvement is not significant. The proposed algorithm retains the idea of the components of the baseline algorithms that perform well for performance improvement (CNN, Residual Network, ConvLSTM, capture of closeness, period, trend), and in addition, other modules are added (SE module, Multi-Attention module, DeConvs). Finally, the data in the Table also verifies that the SE-MAConvLSTM algorithm has a good capture effect on the potential internal characteristics of traffic data (complex spatio-temporal characteristics, closeness similarity, period similarity, trend similarity).

#### 5.4.3 Variant ablation experiment

In this subsection, we verify the importance of the Residual Network Module and the closeness data module, period data module, and trend data module by adding or reducing the modules in the SE-MAConvLSTM framework.

Validity of Residual Connection:

1. SE-MAConvLSTM: Its details are in Section 4 of this paper.
2. SE-MAConvLSTM(No ResNet): We reduce the Residual connections compared to SE-MAConvLSTM.

**Fig 8:**
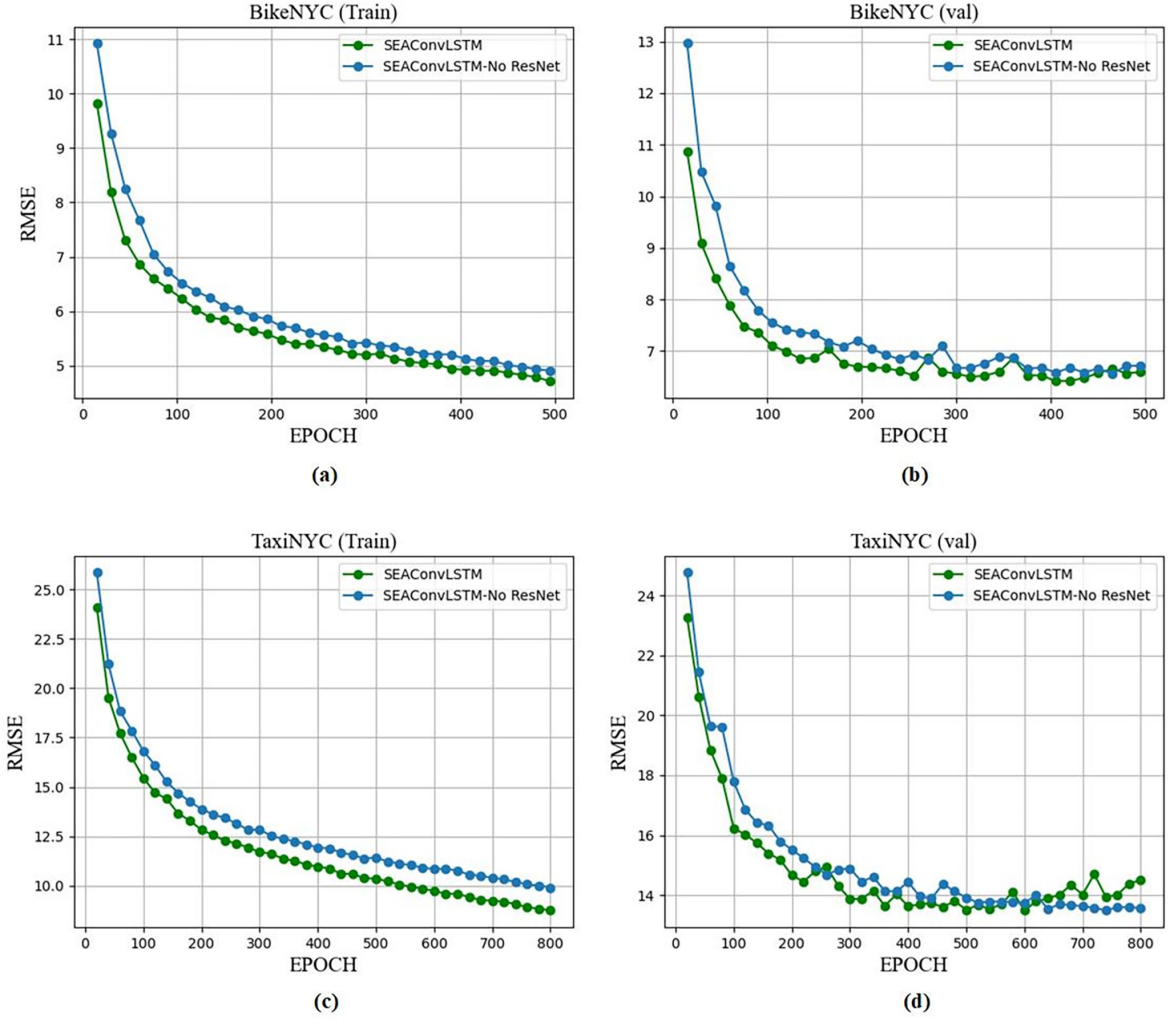
Training process and validation process of SE-MAConvLSTM and SE-MAConvLSTM (No ResNet) on two datasets. (a)BikeNYC(Train) (b)BikeNYC(Val) (c)TaxiNYC(Train) (d)TaxiNYC(Val).

Process of Convergence:

Fig 8 shows the RMSE curve of the training phase. It can be seen that the SE-MAConvLSTM algorithm and the SE-MAConvLSTM(No ResNet) algorithm have achieved convergence on both datasets (dataset TaxiNYC and dataset BikeNYC).

Test Set Performance Comparison:

The test performance of SE-MAConvLSTM and SE-MAConvLSTM(No ResNet) on the two datasets is shown in Table 2. It can be seen that the SE-MAConvLSTM algorithm has better performance than the SE-MAConvLSTM(No ResNet) algorithm on the two datasets, and the performance improvement is 1.5% and 2.5% respectively, indicating that the use of residual connections can help improve the competitiveness of the model.

**Table 2:** Test performance of SE-MAConvLSTM and SE-MAConvLSTM(No ResNet) on two datasets.

Model	TaxiNYC	BikeNYC
SE-MAConvLSTM(No ResNet)	11.647	6.464
SE-MAConvLSTM	11.475	6.302

Closeness, Period, Trend:

1. SE-MAConvLSTM: Its details are in Section 4 of this paper.
2. SE-MAConvLSTM-CP: Compared with SE-MAConvLSTM, we remove trend data and trend attention mechanism.
3. SE-MAConvLSTM-C: Compared with SE-MAConvLSTM-CP, we remove the period and trend data and the corresponding attention mechanism.

**Fig 9:**
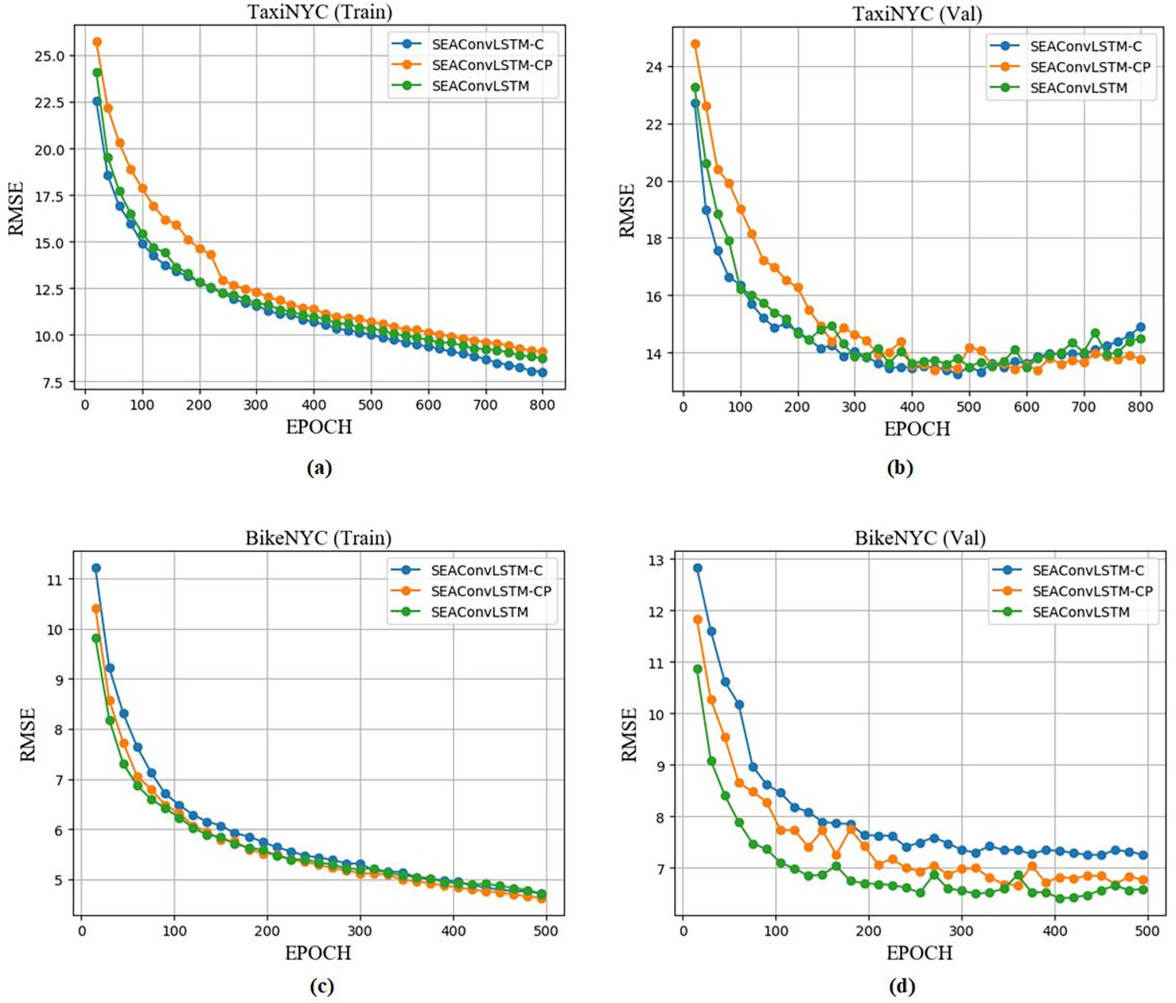
The training process and validation process of SE-MAConvLSTM and its variants on two datasets. (a)TaxiNYC(Train) (b)TaxiNYC(Val) (c)BikeNYC(Train) (d)BikeNYC(Val).

Process of Convergence:

Fig 9 shows the training phase of the SE-MAConvLSTM model and its variants, and it can be seen from the training set and validation set that the model has achieved convergence.

Performance comparison:

The test performance of SE-MAConvLSTM and its variants on the two datasets is shown in Table 3, It shows the performance comparison between SE-MAConvLSTM and variants on the test set. Compared with SE-MAConvLSTM-C, the performance (RMSE) improvement of SE-MAConvLSTM-CP is 1.2% and 6.5% on two datasets (TaxiNYC and BikeNYC) respectively, which proves that adding period data and period attention module can increase the capture ability of model features. SE-MAConvLSTM achieves an improvement (1.1%/1.7%) over SE-MaconVLSTM-CP, which proves that trend data and trend attention modules can help reduce RMSE.

**Table 3:** Test performance of SE-MAConvLSTM and its variants on two datasets.

Model	TaxiNYC	BikeNYC
SE-MAConvLSTM-C	11.74	6.859
SE-MAConvLSTM-CP	11.604	6.412
SE-MAConvLSTM	11.475	6.302

#### 5.4.4 Parameter study

The effect of Reduction parameter in SE Network Module on the result is shown in Table 4, It can be seen that SE-MAConvLSTM algorithm achieves the best performance (11.475/6.302) on both datasets when Reduction parameter is equal to 4. The RMSE is reduced by 1.8% and 4% compared to the worst result.

**Table 4:** Effect of reduction parameter in SE module on the result.

Reduction	TaxiNYC	BikeNYC
2	11.688	6.511
4	11.475	6.302
8	11.528	6.564

#### 5.4.5 Comparison of model parameters

DeepSTN+ is the best among the baseline models. We choose it to compare with the SE-MAConvLSTM model, and Table 5 shows that the number of parameters of the SE-MAConvLSTM model is 1% of the DeepSTN+ model.

**Table 5:** Comparison of model parameters.

Model	TaxiNYC	BikeNYC
DeepSTN+	20519858	32554482
SE-MAConvLSTM	485954	485954

#### 5.4.6 Comparison of predicted and true values at the best performance

**Fig 10:**
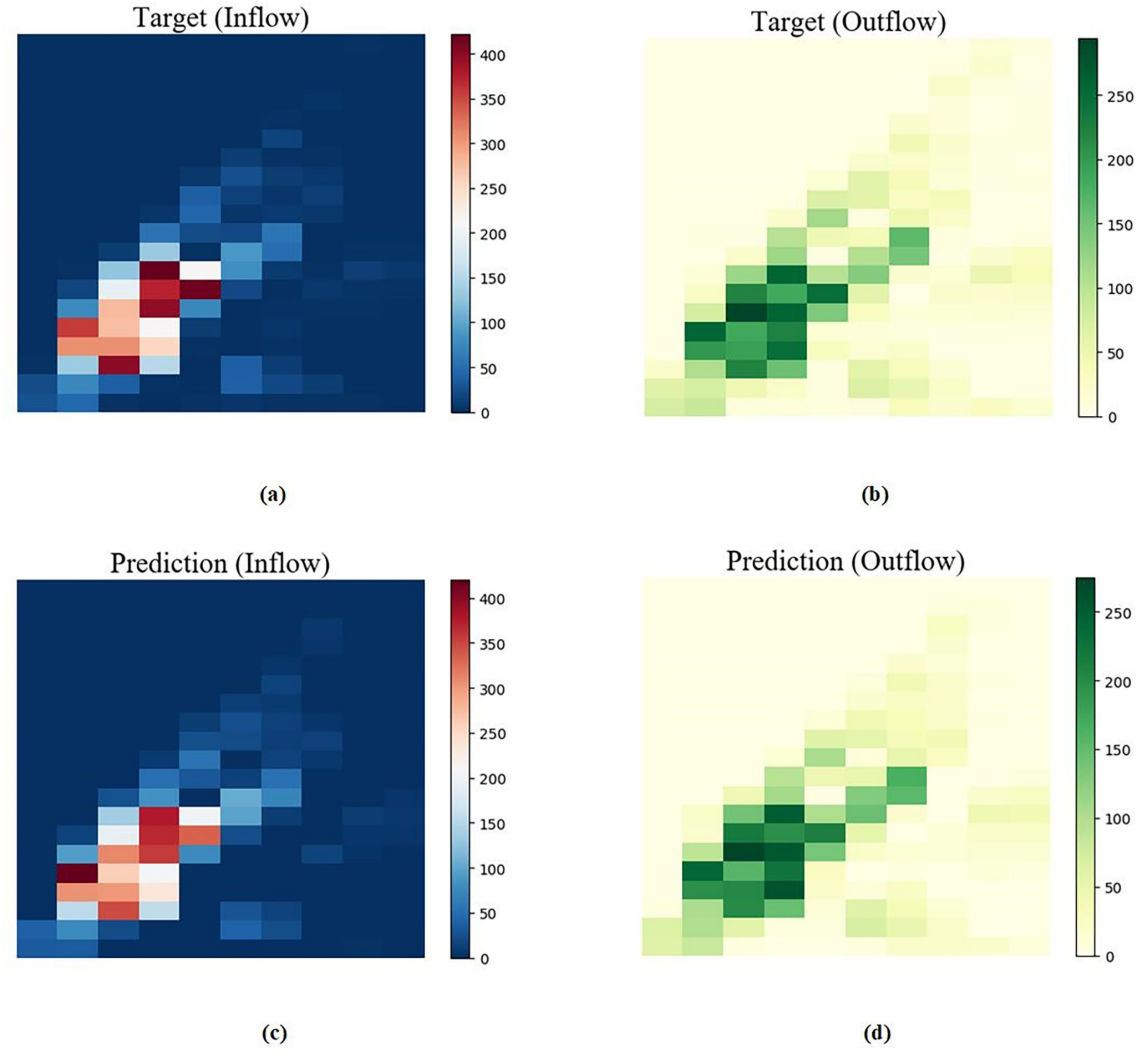
TaxiNYC dataset comparison of predicted and true values for a given time period. (a)Target(Inflow) (b)Target(Outflow) (c)Prediction(Inflow) (d)Prediction(Outflow).

**Fig 11:**
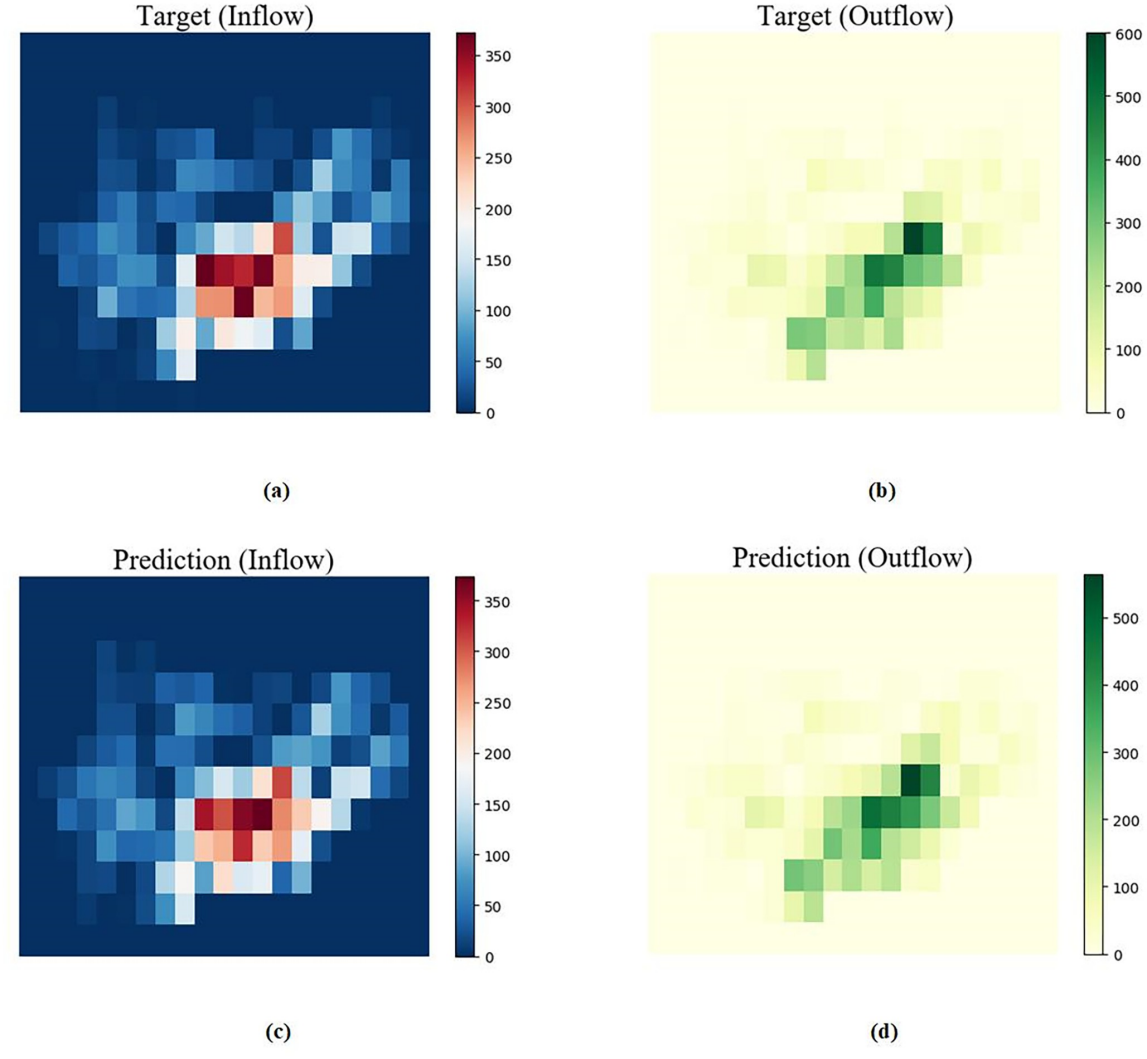
BikeNYC dataset comparison of predicted and real values for a given time period. (a)Target(Inflow) (b)Target(Outflow) (c)Prediction(Inflow) (d)Prediction(Outflow).

The comparison between the predicted effect diagram of our proposed algorithm and the real effect diagram on the two data sets is shown in Figs 10 and 11. It can be seen that the density distribution is very similar, indicating the effectiveness of our proposed model.

## 6 Conclusion

The improvement of traffic flow prediction accuracy depends on the following aspects. Firstly, the degree of spatio-temporal complexity capture. Secondly, the degree of obtaining the characteristics of traffic data (proximity, periodicity, trend). Third, the proportion of each channel in the same time interval data. To address the above issues, we developed a deep learning framework based on Squeeze-and-Excitation Network and Multi-Attention Convolutional LSTM Network. The SE-MAConvLSTM algorithm consists of STREM, Multi-Attention Module and Fusion Module. STFEM helps to better capture the spatio-temporal characteristics of traffic data, and the Squeeze-and-Excitation Network in it helps to automatically assign weights to the inflow matrix and outflow matrix. The Multi-Attention Module is used to capture the traffic data features, and the Fusion Module is used to fuse a series of outputs to obtain the prediction value. Experimental results on two data sets show that the model has certain advantages in improving traffic flow prediction, and can effectively capture the complex nonlinear characteristics of traffic flow over time. In addition, traffic flow is affected by many other external factors, such as the number of lanes, traffic accidents, extreme weather, and social events, and such issues will be considered in the next step.

## Supporting information

S1 Dataset (ZIP)
